# A Cryptogenic Stroke Associated With Infective Endocarditis and Antiphospholipid Antibody Syndrome: Case Report and Literature Review

**DOI:** 10.3389/fneur.2022.872279

**Published:** 2022-07-25

**Authors:** Lei Chen, Ping Zhang, Xuan Zhu, Minmin Zhang, Benqiang Deng

**Affiliations:** Department of Neurovascular Center, Changhai Hospital, Naval Medical University, Shanghai, China

**Keywords:** antiphospholipid antibody syndrome (APS), infective endocarditis, embolic, cryptogenic stroke, surgery

## Abstract

**Introduction:**

Accurate definition of stroke etiology is crucial, as this will guide effective targets for treatment. Both antiphospholipid antibody syndrome (APS) and infective endocarditis (IE) can be independent risk factors for ischemic stroke in young adults. When an embolic stroke occurs with IE and APS simultaneously, the origin of the embolic source is difficult to identify.

**Case Report:**

A 19-year-old man was admitted to the hospital for the onset of stroke. A diagnosis of APS accompanied by IE was made after a series of examinations. We identified aortic valve vegetation as the embolic source. Although both APS and IE can induce valve vegetation, we considered IE to be the primary cause according to the infective clues. Despite treatment with ampicillin, the patient's fever persisted, and surgical aortic valve replacement was performed urgently. The patient recovered without recurrence of stroke during the 1-year follow-up.

**Conclusion:**

A considerable challenge for physicians is evaluating all the signs suggestive of embolic sources in acute stroke and identifying the primary etiology when there are multiple causes. Early diagnosis and surgical intervention for bicuspid aortic valve (BAV) vegetation complicated by acute stroke may yield favorable clinical results.

## Introduction

Approximately 35% of non-lacunar stroke cases occur due to cardioembolic sources (1). Intracardiac thrombi can indicate various common diseases, such as atrial fibrillation, patent foramen ovale (PFO), papillary fibroelastoma, myxoma, and infective endocarditis (IE) (1). Native valve disease, such as a bicuspid aortic valve (BAV) vegetation-induced septic embolic cerebrovascular accident, is even less common (2). Although rare, antiphospholipid antibody syndrome (APS) can develop into cardiac valvular lesions and produce intracardiac thrombi (3, 4). While cardioembolic stroke is often a severe condition and the etiology is various, diagnosis is challenging for physicians, particularly given the time pressure (5). We report a rare case of stroke in a young patient with BAV vegetation who did not present with any clinical features referable to the cardiovascular system before this attack. This stroke was thought to be cryptogenic because it could be associated with IE and APS at the same time.

## Case Description

**Figure 1:**
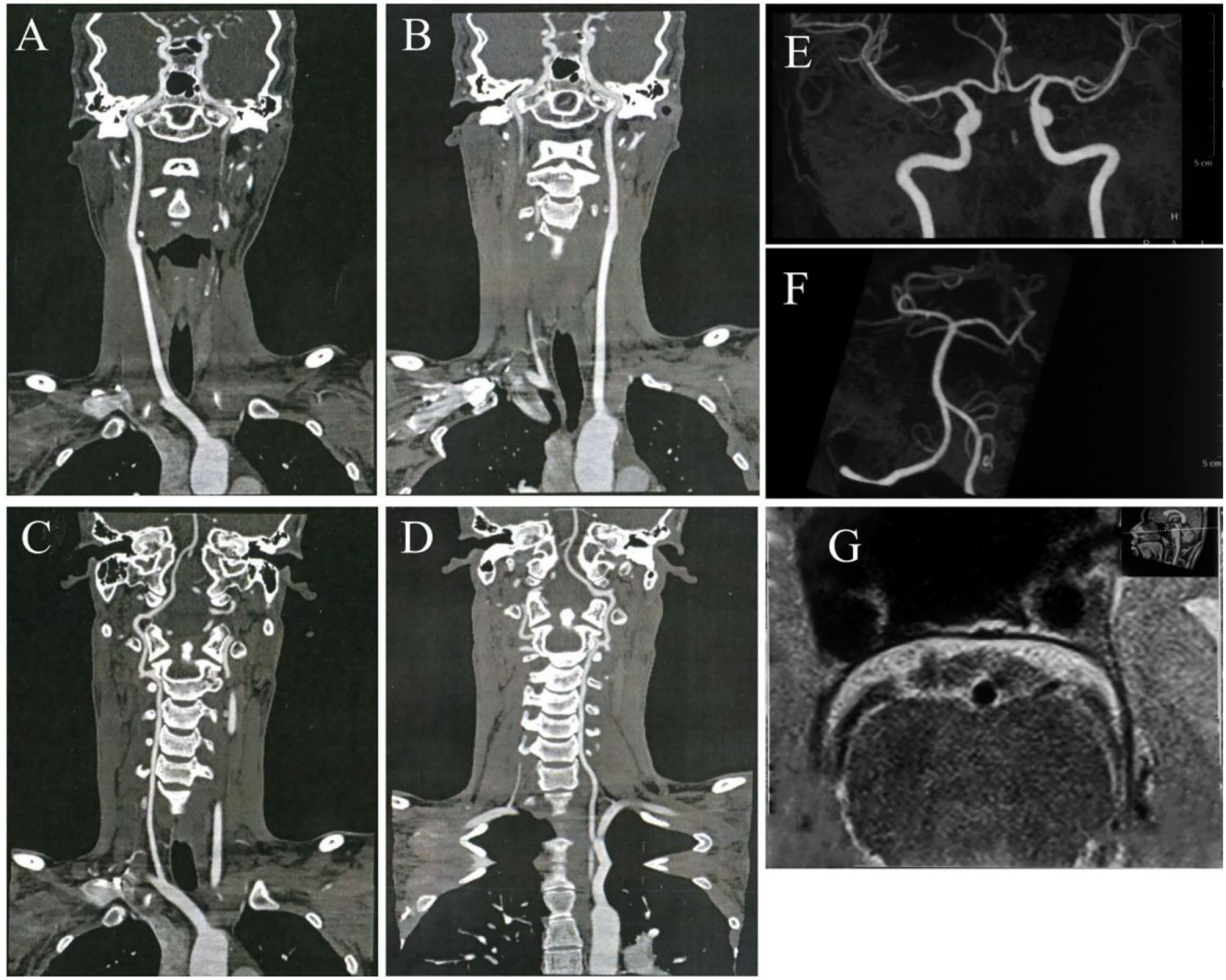
No arterial occlusion, stenosis, or plaque was discovered in bilateral carotid arteries **(A,B)**, vertebral arteries **(C,D)**, and all the intracranial arteries **(E,F)**. The high-resolution vessel wall magnetic resonance imaging (MRI) of the basilar artery shows no occlusion or stenosis **(G)**.

An 18-year-old man was referred to the ER for sudden onset of left hemiplegia, vomiting, and disturbance of consciousness. He was a healthy college student who had never taken any medication before for any disease or illness. There was no exposure to toxins or history of alcohol intake. The patient's family history was significant only for hypertension in his grandmother. A complete system review was negative. His vital signs on admission were as follows: blood pressure, 105/63 mmHg; pulse, 84 beats/min; respiration, 18 breaths/min; and temperature, 36.5°C. Neurological investigation revealed somnolence, global aphasia, gaze palsy, and right-sided hemiplegia. The National Institutes of Health Stroke Scale (NIHSS) score was 15. The patient was transferred to our neurovascular center after 5 h of onset, so thrombolysis with alteplase was not administered. He was not a candidate for acute intervention because multimodal computed tomography revealed no arterial occlusion or perfusion defect (Figure 1), and after this examination, the patient had significant recovery of his consciousness. His power improved to 4/5 in the affected limbs, bringing his NIHSS score to 1. Treatment of aspirin, clopidogrel, and atorvastatin was administrated. Laboratory parameters on admission indicated an acute bacterial infection with a C-reactive protein (CRP) level of 38.21 mg/L and leukocytosis of 12.71 × 10^9^/L.

**Figure 2:**
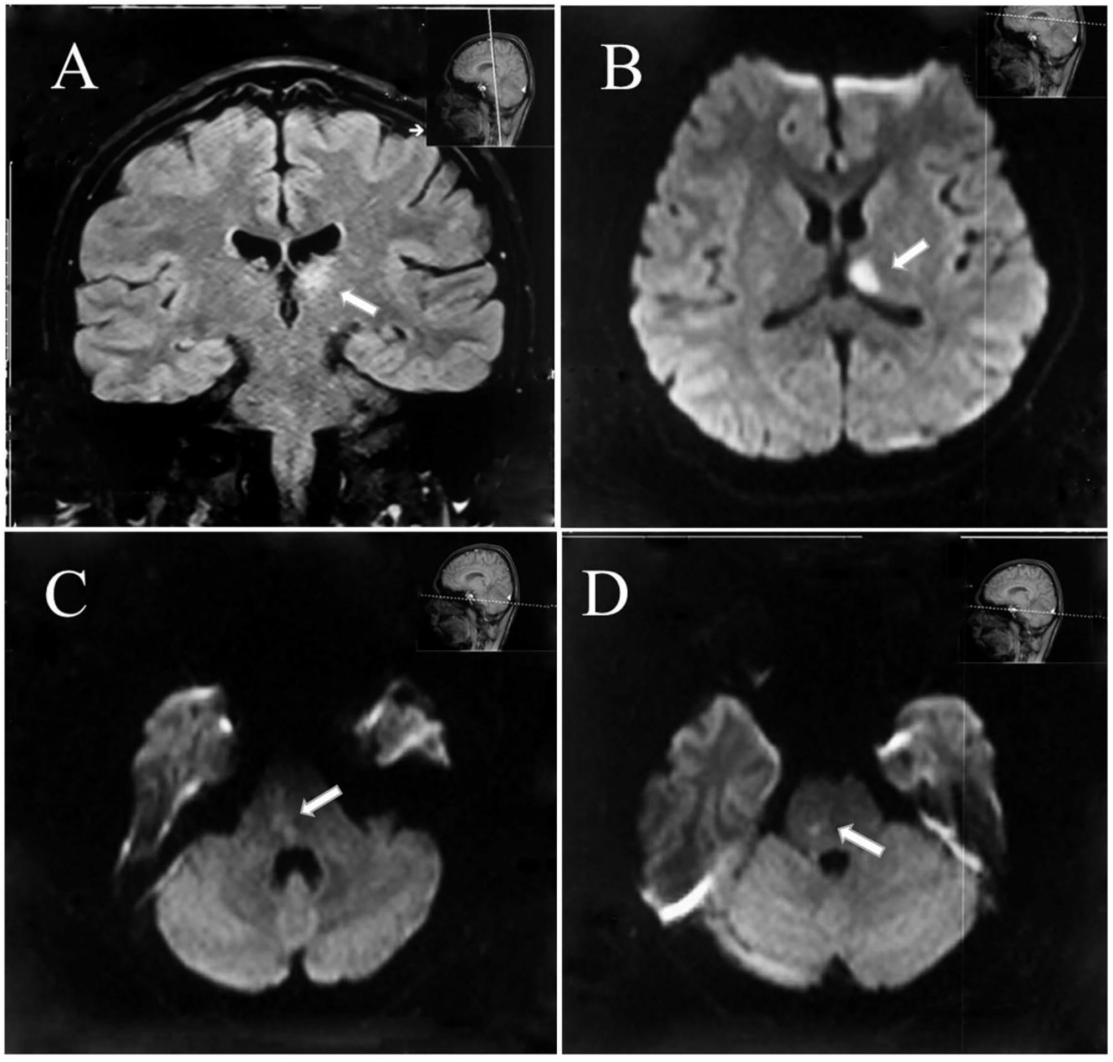
Diffusion restriction of the left thalamus **(A,B)** and the right brain stem as demonstrated on diffusion-weighted magnetic resonance imaging consistent with acute ischemic infarction **(C,D)**.

Neuroimaging with brain magnetic resonance imaging (MRI) showed foci of restricted diffusion in the left thalamus and the right brain stem suggestive of an embolic stroke (Figure 2). Blood work showed an erythrocyte sedimentation rate (ESR) of 38 mm/h and an antistreptolysin O (ASO) concentration of 290.01 IU/ml. The patient tested positive for antiphospholipid (aPL) antibodies, including antibodies against anticardiolipin (aCL) antibodies, lupus anticoagulant (LA), and β2-glycoprotein-1 (β2GP-1). The β2GP-1 (133 relative unit (RU)/ml) level was elevated in high titers. Hence, a diagnosis of APS was considered. At the same time, a transthoracic echocardiogram (TTE) revealed a BAV with moderate regurgitation and vegetation. The vegetation was attached to the anterior commissure, and the longest oscillating mass was 8 mm. Supported by the infection evidence, we believed septic emboli due to IE should be the primary etiology despite APS. However, the patient developed an increasing fever with shivering after 5 days of antibiotic therapy with high-dose penicillin. Further etiological workup on blood cultures demonstrated the growth of oral *Streptococcus*, and the patient was transferred to thoracic surgery for aortic valve replacement. Seven weeks after successful mechanical aortic valve replacement, the patient was discharged with only mild unsteadiness. He received a total 6-week course of IV penicillin in the hospital and was advised to continue a long-term warfarin treatment. The patient did not receive any immunotherapy and his aCL, LA, and β2GP-1 tests were still positive in other hospitals in half a year. He had no residual neurological deficits or recurrence of stoke when evaluated 1 year later. The patient had returned to the college and felt that he can live and study as before.

The timeline of the case is summarized in (Table 1).

**Table 1:** The clinical features and treatment of the patient according to the timeline.

**Clinical features**	**Time**	**Treatment**
Left hemiplegia and left facial numbness	2018-11-17 14:00	
Left hemiplegia, vomiting, and disturbance of consciousness	2018-11-17 17:00	
The patient arrived at the emergency room and presented somnolence, global aphasia, left hemiplegia, gaze palsy, and emesis. (NHISS 15)	2018-11-17 19:00	
Returned consciousness and left hemiparesis (NHISS 1)	2018-11-17 19:30	Aspirin, clopidogrel, and atrovastatin
Antiphospholipid antibodies (+)	2018-11-21	
Transthoracic echocardiogram (TTE) revealed bicuspid aortic valve (BAV) vegetation	2018-11-22	Penicillin and nadroparin calcium (stop aspirin and clopidogrel)
Fever and chills	2018-11-27	
Blood cultures demonstrated the growth of oral *Streptococcus*.	2018-11-28	
There are no additional focal neurological deficits (NIHSS 1). His temperature was normal for 10 days and the repeated brain MRI did not show any new infarct lesions	2018-12-10	Stopped nadroparin calcium 2 days ago, and aortic valve replacement was performed
	2018-12-16	Penicillin and warfarin (add warfarin)
Discharge	2019-01-22	The patient finished 6-week course of penicillin injection and continued warfarin treatment
No residual neurological deficits and no recurrence of stoke	2020-01-31	Warfarin

## Discussion

According to the episode, this patient suffered from acute stroke due to basilar artery occlusion and was soon self-recanalized. Following the assessment, large-artery atherosclerosis and small-vessel occlusion, two of the most common stroke etiologies, were excluded first. The cardioembolic stroke had been considered in the presence of BAV vegetation with multiple bilateral lesions on MRI. BAV is a congenital heart abnormality that may involve endocarditis, which is the most severe comorbidity with significant morbidity (6). Two types of endocarditis, infective and non-infective, can both cause stroke (5). Cardiac embolism due to IE is an extraordinary stroke etiology, accounting for nearly 30% of all patients with IE (7). Non-infective endocarditis can complicate APS, which is a systemic autoimmune disease characterized by thrombotic complications in patients positive for aPL (5, 8, 9). The APS-associated non-infective endocarditis was reported to be Libman–Sacks (LS) endocarditis (10). LS endocarditis can be quite difficult to diagnose and often mimics the presentation of bacterial endocarditis. In this case, IE and APS were found to coexist, but which disease was accountable for the aortic valve vegetation was ambiguous. Treatments of APS-induced stroke are antithrombotic medications and modulation of the immune response with immunotherapy, while IE requires antibiotics or even emergency surgery. The radical etiology of this cardioembolic stroke needed to be identified to inform the treatment.

Management decisions for patients with complicated cardioembolic stroke should be discussed and decided by a multidisciplinary team comprised of cardiologists, infectious diseases specialists, and cardiothoracic surgeons with a major contribution from neurologists. We consulted all the specialists and deemed IE to be the primary cause of the cardiac embolism in terms of the patient's elevated leukocytosis and CRP level as well as the positive blood cultures. In APS, valvular vegetations are often non-bacterial with negative blood cultures (11). These findings were consistent with the evaluation of helpful markers in distinguishing IE from LS endocarditis in the previous literature (10).

In ~15–20% of patients with IE, clinical features referable to the cardiovascular system, such as clubbing, splinter hemorrhages, and hematuria, may be absent (12). Furthermore, if fever is not present during the initial evaluation, a rapid and accurate diagnosis of IE complicated by stroke is difficult during the rapid intravenous thrombolysis (IVT) treatment process in the emergency department. IVT and endovascular therapy (EVT) are the standard of care in selected patients for acute ischemic stroke, but their use in patients with stroke secondary to IE is controversial (13, 14). IVT is contraindicated because of the increased risk of intracranial hemorrhage and worse outcomes (15, 16). We found 10 cases of IE-related acute ischemic stroke who received IVT, and IE was suspected post-thrombolysis in all these cases (17–22). Nine of them were identified with sufficient clinical outcome data (17–19, 21, 22) (Table 2). Among the nine cases, the median age was 56 years (range 25–75 years); four were women and five were men. Vegetations affected the mitral valve in 78% of these cases and the aortic valve in the remainder. Eight of the nine cases reported preprocedure NIHSS scores, but only one reported postprocedure NIHSS scores, so we could not evaluate the improvement in NIHSS scores. The median modified Ranking Scale (mRS) was 3, and only two patients achieved good functional outcomes (mRS≤2). Of all these reported cases, 56% (5/9) developed symptomatic intracranial hemorrhage (sICH), and the mortality reached 44% (4/9). IE should be considered from the outset to avoid IVT, particularly given the time pressures in acute stroke care.

**Table 2:** Summary of clinical characteristics in reported case series of IE presenting as AIS treated with IVT.

**Reference**	**Age/Sex**	**Affected valve**	**Acute treatment**	**NIHSS (initial)**	**NIHSS (follow up)**	**mRS**	**sICH**	**Mortality**
Ashkanani et al. (17)	65/M	Mitral valve	IVT	18	ND	3 (8 weeks)	No	No
Brownlee et al. (18)	27/F	Mitral valve	IVT	15	20	1 (6 months)	Yes	No
Gopal et al. (19)	44/F	Mitral valve	IVT	4	ND	6 (3 months)	No	Yes
Gopal et al. (19)	56/F	Mitral valve	IVT	2	ND	6 (3 months)	No	Yes
Gopal et al. (19)	74/F	Mitral valve	IVT	8	ND	3 (3 months)	No	No
Gopal et al. (19)	66/M	Mitral valve	IVT	7	ND	6 (3 months)	Yes	Yes
Gopal et al. (19)	25/M	Mitral valve	IVT	3	ND	3 (3 months)	Yes	No
Maeoka et al. (21)	46/M	Aortic valve	IVT + EVT	ND	ND	1 (ND)	Yes	No
Distefano et al. (22)	75/M	Aortic valve	IVT + EVT	16	ND	6 (ND)	Yes	Yes

*IE, infective endocarditis; AIS, acute ischemic stroke; IVT, intravenous thrombolysis; NIHSS, National Institutes of Health Stroke Scale; mRS, modified Ranking Scale; sICH, symptomatic intracranial hemorrhage; F, female; M, male; EVT, endovascular therapy; ND, not described*.

Endovascular therapy (EVT) may be a choice in ischemic stroke with large vessel occlusion due to cardiac emboli. Routine histopathological analysis of EVT-retrieved clots could have value in confirming clinical diagnoses and supporting early treatment in acute stroke with IE (23, 24). Even though EVT results in highly successful recanalization rates, stroke patients with IE are most likely to have worse clinical and safety outcomes than non-IE patients following reperfusion therapy in the acute phase (25, 26). With the limitation of the available evidence and a lack of consensus, EVT still should be considered seriously as the efficacy and safety have not yet been well established (14).

In many cases of IE, vegetation decreases during treatment with antibiotics. However, in some patients, valvular lesions that form the basis for the development of IE may remain after treatment with antibiotics (27). Surgery is recommended in uncontrolled infections according to the European Society of Cardiology (ESC) guidelines (15). Due to the persistent fever and burdensome BAV vegetations, valve surgery seemed to be an imminent emergency in our patient. However, the benefit of potential valve surgery can be challenging given the risk of bleeding in the setting of high doses of heparin during surgery and worsening neurological deficits attributed to perioperative hypotension in IE patients who have already had a stroke (28). Traditionally, cardiac surgery should be delayed for several months in endocarditis complicated by stroke, while a growing body of available evidence supports that early surgery is beneficial for outcomes (29, 30). In this patient, the surgery was performed 24 days after stroke onset as the infection could not be controlled by antibiotics. Postponing surgery to achieve clinical stabilization and better perioperative circumstances may have worsened the disease process with recurrent embolization and resulted in heart failure. Early surgery for IE with small acute cerebral infarction (<2 cm) was confirmed to be performed safely with good outcomes (31). As the stroke lesions were small with a low risk of hemorrhage, this patient eventually recovered well after the early surgery.

### Limitation

Aspirin has negligible or poor effects and shows a heightened risk of hemorrhage in IE-related acute ischemic stroke; therefore, the use of aspirin is not recommended in the early therapy of patients with IE (32). Fortunately, the delay in IE diagnosis and the use of aspirin and clopidogrel did not result in hemorrhage complications in this case. A 5-year prospective study showed that the median time for a recurrent thrombotic event is shorter in patients with a high titer of aCL antibodies and intracardiac thrombus (33). This patient did not receive any immunotherapy, and his aPL level remained high. Furthermore, we still need long-term follow-up to confirm the diagnosis. This literature review may also have lapsed due to publication bias.

## Conclusion

Bicuspid aortic valve (BAV) vegetation-related cerebral embolism may present as cryptogenic and can be confusing in the acute phase, particularly when APS and IE are diagnosed simultaneously. Early consultation with a multidisciplinary team can be extremely helpful. Timely surgical intervention should always be considered in cases of small cerebral infarction accompanied by large valve vegetations. Going forward, there is a demand for further study within this area to direct future practice.

## Data Availability Statement

The original contributions presented in the study are included in the article/supplementary material, further inquiries can be directed to the corresponding author.

## Ethics Statement

The study involving human participants was reviewed and approved by Shanghai Changhai Hosptial Ethics Committee. Written informed consent was obtained from the individual(s) for the publication of any potentially identifiable images or data included in this article.

## Author Contributions

LC and PZ: conceptualization and writing of the article. XZ and MZ: original case and draft preparation. BD: supervision and reviewing. All authors contributed to the article and approved the submitted version.

## Conflict of Interest

The authors declare that the research was conducted in the absence of any commercial or financial relationships that could be construed as a potential conflict of interest.

## Publisher's Note

All claims expressed in this article are solely those of the authors and do not necessarily represent those of their affiliated organizations, or those of the publisher, the editors and the reviewers. Any product that may be evaluated in this article, or claim that may be made by its manufacturer, is not guaranteed or endorsed by the publisher.
